# [μ-3-(8-Quinol­yloxy)propanedi­yl]bis­[dicarbon­yl(η^5^-methoxy­carbonyl­cyclo­penta­dien­yl)molybdenum(III)]

**DOI:** 10.1107/S1600536810007683

**Published:** 2010-03-06

**Authors:** Ai-Ling Guo, Mei Zhao, Jian-Ping Ma, Dian-Shun Guo

**Affiliations:** a Department of Chemistry, Shandong Normal University, Jinan 250014, People’s Republic of China

## Abstract

The crystal structure of the title dimolybdenum complex, [Mo_2_(C_12_H_9_NO)(C_7_H_7_O_2_)_2_(CO)_4_], has a quasi-tetra­hedral Mo_2_C_2_ cluster core attached to one 3-(8-quinol­yloxy)propanediyl (*L*) and two methoxy­carbonyl­cyclo­penta­dienyl (Cp’) ligands which are coordinated to two Mo atoms: one Mo atom bonds two terminal CO ligands while the other links one terminal and one semi-bridging CO ligand. An intra­molecular C—H⋯N hydrogen bond results in the quinolyl plane of the *L* ligand approaching and being nearly perpendicular to one of the Cp’ rings [88.09 (12)°]. In the supra­molecular structure, a one-dimensional comb-shaped infinite chain is formed approximately along the crystallographic *c* axis by a combination of inter­molecular C—H⋯O hydrogen bonds and locally generates a *C*(6) motif. Finally, pairs of inversion-related comb-shaped chains associate into a new ladder-shaped infinite chain through weak π–π stacking inter­actions between neighbouring quinolyl systems (pyridyl centroid–centroid distance = 3.853 Å).

## Related literature

For general background to dimolybdenum alkyne complexes, see: Conole *et al.* (1989 ▶, 1990 ▶); Adams *et al.* (1995 ▶, 1996 ▶); Muetterties (1980 ▶); Gibson *et al.* (1991 ▶); Brady & Pettit (1980 ▶); Sappa *et al.* (1983 ▶, 1985 ▶); Raithby & Rosales (1985 ▶). For semi-bridging CO ligands, see: Curtis & Butler (1978 ▶); Klingler *et al.* (1978 ▶). For related structures, see: Bailey *et al.* (1978 ▶); Song *et al.* (1996 ▶); Zhang *et al.* (1999 ▶). For hydrogen-bond motifs, see: Bernstein *et al.* (1995 ▶). For the synthesis, see: Birdwhistell *et al.* (1978 ▶).
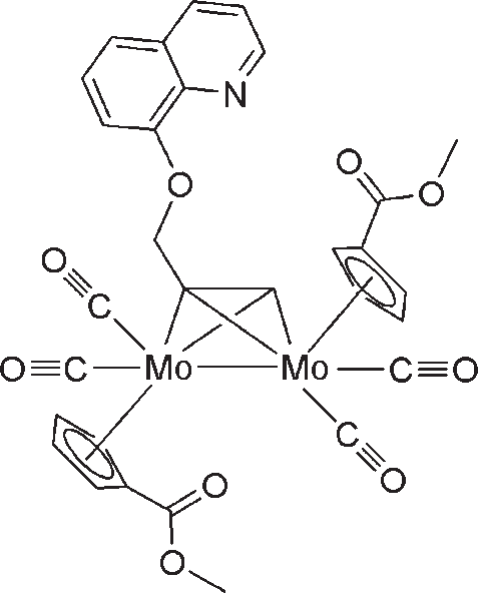


## Experimental

### 

#### Crystal data

[Mo_2_(C_12_H_9_NO)(C_7_H_7_O_2_)_2_(CO)_4_]
*M* *_r_* = 733.37
Monoclinic, 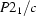

*a* = 7.744 (1) Å
*b* = 12.7189 (16) Å
*c* = 28.630 (3) Å
β = 100.012 (3)°
*V* = 2777.0 (6) Å^3^
*Z* = 4
Mo *K*α radiation
μ = 0.96 mm^−1^
*T* = 298 K
0.42 × 0.18 × 0.04 mm

#### Data collection

Bruker SMART CCD area-detector diffractometer
Absorption correction: multi-scan (*SADABS*; Bruker, 1999 ▶) *T*_min_ = 0.688, *T*_max_ = 0.963
14074 measured reflections
5071 independent reflections
4445 reflections with *I* > 2σ(*I*)
*R*_int_ = 0.032

#### Refinement

*R*[*F* ^2^ > 2σ(*F* ^2^)] = 0.039
*wR*(*F* ^2^) = 0.094
*S* = 1.06
5071 reflections
381 parameters
H-atom parameters constrained
Δρ_max_ = 0.54 e Å^−3^
Δρ_min_ = −0.41 e Å^−3^

### 

Data collection: *SMART* (Bruker, 1999 ▶); cell refinement: *SAINT* (Bruker, 1999 ▶); data reduction: *SAINT*; program(s) used to solve structure: *SHELXS97* (Sheldrick, 2008 ▶); program(s) used to refine structure: *SHELXL97* (Sheldrick, 2008 ▶); molecular graphics: *SHELXTL* (Sheldrick, 2008 ▶); software used to prepare material for publication: *SHELXTL*.

## Supplementary Material

Crystal structure: contains datablocks I, global. DOI: 10.1107/S1600536810007683/bg2333sup1.cif

Structure factors: contains datablocks I. DOI: 10.1107/S1600536810007683/bg2333Isup2.hkl

Additional supplementary materials:  crystallographic information; 3D view; checkCIF report

## Figures and Tables

**Table 1:** Hydrogen-bond geometry (Å, °)

*D*—H⋯*A*	*D*—H	H⋯*A*	*D*⋯*A*	*D*—H⋯*A*
C14—H14⋯N1	0.98	2.61	3.344 (6)	132
C15—H15⋯O4^i^	0.98	2.36	3.191 (5)	143

Symmetry code: (i) 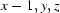.

**Fig. 1.**
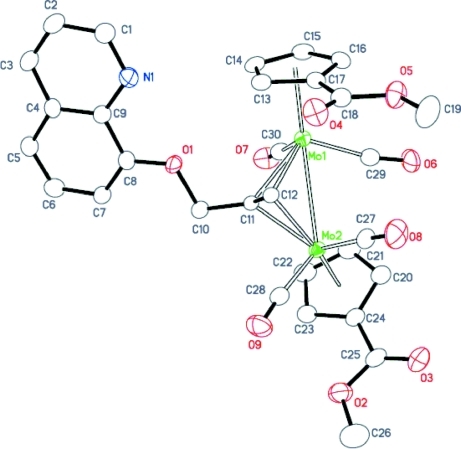
The molecular structure of the title compound with displacement ellipsoids drawn at the 30% probability level for non-H atoms.

**Fig. 2.**
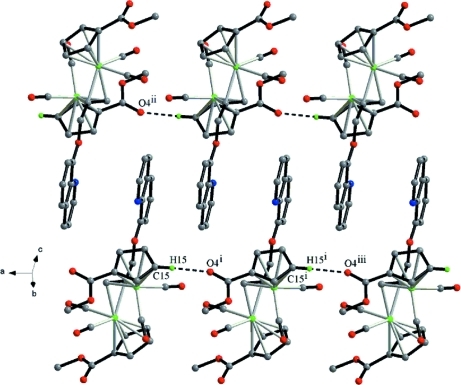
A view of the hydrogen-bonded ladder-shaped chain of the title molecule approximatively along the c axis. Some hydrogen atoms are omitted for clarity. [Symmetry codes: (i) x - 1, y, z; (ii) -x + 1, -y + 1, -z + 2; (iii) x - 2, y, z].

## supplementary crystallographic information

### Comment

The dimolybdenum alkyne complexes have been extensively examined (Conole *et al.*, 1989, 1990; Adams *et al.*, 1995, 1996) due to their potential applications in catalysis and materials (Muetterties, 1980; Gibson *et al.*, 1991; Brady & Pettit, 1980). Moreover, such complexes can be used as precursors for constructing higher-nuclearity alkyne clusters (Sappa *et al.*, 1983, 1985; Raithby & Rosales, 1985). While there is no example of using these clusters as building blocks for the construction of supramolecular assemblies. We report here a novel ladder-shaped chain sructure composed of a new dimolybdenum alkyne complex, Mo_2_Cp'_2_(µ-L)(CO)_4_ [Cp' = *η*^5^-C_5_H_4_CO_2_CH_3_, L = 8-(2-propynyloxy)quinoline], namely µ-[3-(8-quinolyloxy)propyne]-dicarbomethoxycyclopentadienyl-tetracarbonyldimolybdenum.

The crystal structure of the title complex, as shown in Fig. 1, contains a quasi-tetrahedral Mo_2_C_2_ cluster core linked to one L and two Cp' ligands which are coordinated to two Mo atoms, respectively, one Mo atom bonded two terminal CO ligands while the other attached one terminal and one semibridging CO ligand (Curtis & Butler 1978; Klingler *et al.*, 1978). The complex has the same basic structure in which there is a crosswise acetylene bridge typical of that found in the related dimolybdenum alkyne compounds (Bailey *et al.*, 1978; Song *et al.*, 1996; Zhang *et al.*, 1999). In the Mo_2_C_2_ cluster core, the Mo—Mo and C—C bond lengths are 2.978 (5) and 1.351 (5) A°, respectively, very close to the corresponding values observed in the cluster [Mo_2_Cp'_2_(µ-C_2_H_2_)(CO)_4_] (Song *et al.*, 1996). Notably, the quinolyl plane belonging to L ligand is more approachable and nearly perpendicular to one Cp' ring, with an interplane angle of 88.09 (12)°, presumedly ascribed to the formation of an intramolecular C14—H14···N1 hydrogen bond (Table 1) between the cyclopentadienyl and quinolyl groups. Such an orientation of the quinolyl plane is different from that of the phenyl ring in the crystal structure of the compound [Mo_2_Cp'_2_(µ-C_2_HPh)(CO)_4_] (Zhang *et al.*, 1999).

In the supramolecular structures of the title cluster, a novel one-dimensional comb-shaped infinite chain is formed approximatively along the crystallographic *c* axis via a combination of intermolecular C—H···O hydrogen bonds (Table 1), locally creating a *C*(6) motif (Bernstein *et al.*, 1995) at each link. The motif arises from atoms C15—H15 in the molecule at (x, y, z), acting as a hydrogen-bond donor to atom O4 at (x - 1, y, z). Pairs of the inversion-related comb-shaped chains associate into a new ladder-shaped infinite chain (Fig. 2), in which there are weak π–π stacking between the neibouring quinolyl rings.

### Experimental

All reactions and manipulations were carried out under dry argon and using standard Schlenk techniques. A solution of [Mo_2_Cp'_2_(CO)_6_,Cp' = *η*^5^-C_5_H_4_CO_2_CH_3_], prepared according to the literature method (Birdwhistell *et al.*, 1978), (0.400 g, 0.66 mmol) in toluene (20 ml) was refluxed for 24 h with a stream of argon bubbling slowly through it to form [Mo_2_Cp'_2_(CO)_4_]. Upon cooling to room temperature, 3-(8-quinolyloxy)propyne (0.605 g, 3.30 mmol) was added to the solution, followed stirring for an additional 2 h. After removal of the volatile under reduced pressure, the residue was separated by flash column chromatography (ethyl acetate/hexane = 1:1, *R*_f_ = 0.5), giving the title compound (yield 35%) as dark red solid. ^1^H NMR (300 MHz, CDCl_3_): *δ* 8.91 (br, 1H), 8.13 (d, 1H, *J* = 8.17 Hz), 7.40–7.48 (m, 3H), 7.11 (d, 1H, *J* = 7.16 Hz), 6.11 (s, 1H), 5.90 (s, 2H), 5.72 (s, 2H), 5.54 (s, 2H), 5.48 (s, 2H), 5.36 (s, 2H), 3.74 (s, 6H). Single crystals of the title compond suitable for X-ray diffraction analysis were obtained by slow evaporation of a solution in hexane and CH_2_Cl_2_ at 298 K.

### Refinement

All non-hydrogen atoms were refined with anisotropic displacement parameters. Hydrogen atoms attached to refined atoms were placed in geometrically idealized positions and refined using a riding model, with C—H = 0.93, 0.98 and 0.97?Å for aromatic, methylene and methyl H, respectively, and Uiso(H) = 1.5Ueq(C) for methyl H, and Uiso(H) = 1.2Ueq(C) for all other H atoms. .

### Crystal data

**Table:**

[Mo_2_(C_12_H_9_NO)(C_7_H_7_O_2_)_2_(CO)_4_]	*F*(000) = 1464
*M**_r_* = 733.37	*D*_x_ = 1.754 Mg m^−^^3^
Monoclinic, *P*2_1_/*c*	Mo *K*α radiation, λ = 0.71073 Å
Hall symbol: -P 2ybc	Cell parameters from 5150 reflections
*a* = 7.744 (1) Å	θ = 2.2–27.6°
*b* = 12.7189 (16) Å	µ = 0.96 mm^−^^1^
*c* = 28.630 (3) Å	*T* = 298 K
β = 100.012 (3)°	Plan, red
*V* = 2777.0 (6) Å^3^	0.42 × 0.18 × 0.04 mm
*Z* = 4	

### Data collection

**Table:**

Bruker SMART CCD area-detector diffractometer	5071 independent reflections
Radiation source: fine-focus sealed tube	4445 reflections with *I* > 2σ(*I*)
graphite	*R*_int_ = 0.032
phi and ω scans	θ_max_ = 25.4°, θ_min_ = 1.8°
Absorption correction: multi-scan (*SADABS*; Bruker, 1999)	*h* = −9→9
*T*_min_ = 0.688, *T*_max_ = 0.963	*k* = −15→11
14074 measured reflections	*l* = −27→34

### Refinement

**Table:**

Refinement on *F*^2^	Primary atom site location: structure-invariant direct methods
Least-squares matrix: full	Secondary atom site location: difference Fourier map
*R*[*F*^2^ > 2σ(*F*^2^)] = 0.039	Hydrogen site location: inferred from neighbouring sites
*wR*(*F*^2^) = 0.094	H-atom parameters constrained
*S* = 1.06	*w* = 1/[σ^2^(*F*_o_^2^) + (0.0485*P*)^2^ + 1.4813*P*] where *P* = (*F*_o_^2^ + 2*F*_c_^2^)/3
5071 reflections	(Δ/σ)_max_ = 0.002
381 parameters	Δρ_max_ = 0.54 e Å^−^^3^
0 restraints	Δρ_min_ = −0.41 e Å^−^^3^

### Special details

**Table:**

Geometry. All esds (except the esd in the dihedral angle between two l.s. planes) are estimated using the full covariance matrix. The cell esds are taken into account individually in the estimation of esds in distances, angles and torsion angles; correlations between esds in cell parameters are only used when they are defined by crystal symmetry. An approximate (isotropic) treatment of cell esds is used for estimating esds involving l.s. planes.
Refinement. Refinement of *F*^2^ against ALL reflections. The weighted *R*-factor wR and goodness of fit *S* are based on *F*^2^, conventional *R*-factors *R* are based on *F*, with *F* set to zero for negative *F*^2^. The threshold expression of *F*^2^ > σ(*F*^2^) is used only for calculating *R*-factors(gt) etc. and is not relevant to the choice of reflections for refinement. *R*-factors based on *F*^2^ are statistically about twice as large as those based on *F*, and *R*- factors based on ALL data will be even larger.

### Fractional atomic coordinates and isotropic or equivalent isotropic displacement parameters (Å^2^)

**Table:**

	*x*	*y*	*z*	*U*_iso_*/*U*_eq_	
Mo1	0.22482 (4)	0.85534 (3)	0.887331 (11)	0.02978 (11)	
Mo2	0.29551 (4)	0.80787 (3)	0.790559 (11)	0.03177 (11)	
O1	0.2230 (4)	0.5812 (2)	0.90476 (9)	0.0521 (8)	
O2	0.4274 (4)	0.7577 (3)	0.66998 (11)	0.0634 (9)	
O3	0.4369 (6)	0.9323 (3)	0.67758 (13)	0.0831 (12)	
O4	0.7408 (4)	0.9014 (3)	0.94201 (12)	0.0563 (8)	
O5	0.6097 (4)	1.0562 (3)	0.92403 (11)	0.0552 (8)	
O6	0.1261 (5)	1.0549 (3)	0.82612 (12)	0.0675 (10)	
O7	−0.1432 (4)	0.7587 (3)	0.85410 (11)	0.0651 (10)	
O8	0.6047 (5)	0.9704 (3)	0.80995 (13)	0.0737 (10)	
O9	0.5944 (5)	0.6569 (3)	0.77104 (12)	0.0663 (10)	
N1	0.2771 (5)	0.5592 (3)	0.99779 (13)	0.0569 (10)	
C1	0.3036 (7)	0.5466 (4)	1.04418 (16)	0.0673 (15)	
H1	0.3452	0.6037	1.0631	0.081*	
C2	0.2727 (7)	0.4519 (4)	1.06671 (16)	0.0629 (14)	
H2	0.2944	0.4472	1.0996	0.075*	
C3	0.2119 (6)	0.3686 (4)	1.04036 (15)	0.0519 (12)	
H3	0.1902	0.3058	1.0549	0.062*	
C4	0.1807 (5)	0.3764 (3)	0.99026 (15)	0.0431 (10)	
C5	0.1187 (6)	0.2923 (3)	0.96027 (16)	0.0505 (11)	
H5	0.1008	0.2267	0.9729	0.061*	
C6	0.0852 (6)	0.3076 (4)	0.91262 (16)	0.0543 (12)	
H6	0.0404	0.2523	0.8929	0.065*	
C7	0.1163 (6)	0.4037 (3)	0.89266 (15)	0.0454 (10)	
H7	0.0920	0.4117	0.8599	0.054*	
C8	0.1818 (5)	0.4864 (3)	0.92034 (14)	0.0418 (10)	
C9	0.2151 (5)	0.4743 (3)	0.97089 (14)	0.0406 (9)	
C10	0.1965 (6)	0.5988 (3)	0.85422 (13)	0.0436 (10)	
H10A	0.0738	0.5910	0.8403	0.052*	
H10B	0.2651	0.5496	0.8391	0.052*	
C11	0.2567 (5)	0.7091 (3)	0.84888 (12)	0.0347 (9)	
C12	0.4110 (5)	0.7593 (3)	0.86228 (12)	0.0316 (8)	
H12	0.5285	0.7351	0.8765	0.038*	
C13	0.4045 (5)	0.8172 (3)	0.96022 (13)	0.0381 (9)	
H13	0.4893	0.7595	0.9660	0.046*	
C14	0.2296 (6)	0.8126 (4)	0.96709 (13)	0.0436 (10)	
H14	0.1720	0.7521	0.9790	0.052*	
C15	0.1585 (5)	0.9153 (4)	0.95922 (13)	0.0432 (10)	
H15	0.0422	0.9373	0.9645	0.052*	
C16	0.2892 (5)	0.9827 (3)	0.94779 (13)	0.0391 (9)	
H16	0.2795	1.0590	0.9436	0.047*	
C17	0.4428 (5)	0.9215 (3)	0.94789 (12)	0.0336 (8)	
C18	0.6130 (5)	0.9562 (3)	0.93762 (13)	0.0398 (10)	
C19	0.7730 (6)	1.0965 (5)	0.91356 (19)	0.0720 (16)	
H19A	0.8632	1.0886	0.9409	0.108*	
H19B	0.7596	1.1695	0.9053	0.108*	
H19C	0.8047	1.0580	0.8875	0.108*	
C20	0.1446 (6)	0.9155 (4)	0.73296 (14)	0.0483 (11)	
H20	0.1626	0.9912	0.7298	0.058*	
C21	0.0138 (6)	0.8680 (4)	0.75394 (15)	0.0517 (12)	
H21	−0.0691	0.9056	0.7702	0.062*	
C22	0.0149 (6)	0.7593 (4)	0.74664 (14)	0.0522 (12)	
H22	−0.0666	0.7083	0.7565	0.063*	
C23	0.1485 (6)	0.7372 (4)	0.72051 (13)	0.0460 (10)	
H23	0.1719	0.6685	0.7074	0.055*	
C24	0.2290 (6)	0.8343 (4)	0.71115 (13)	0.0448 (11)	
C25	0.3747 (6)	0.8499 (4)	0.68456 (14)	0.0518 (12)	
C26	0.5796 (8)	0.7607 (5)	0.6472 (2)	0.0825 (17)	
H26A	0.5589	0.8086	0.6209	0.124*	
H26B	0.6018	0.6917	0.6360	0.124*	
H26C	0.6794	0.7840	0.6695	0.124*	
C27	0.4917 (5)	0.9115 (3)	0.80390 (14)	0.0427 (10)	
C28	0.4856 (6)	0.7096 (3)	0.77944 (14)	0.0415 (10)	
C29	0.1655 (5)	0.9754 (4)	0.84529 (14)	0.0430 (10)	
C30	−0.0102 (5)	0.7963 (3)	0.86565 (14)	0.0428 (10)	

### Atomic displacement parameters (Å^2^)

**Table:**

	*U*^11^	*U*^22^	*U*^33^	*U*^12^	*U*^13^	*U*^23^
Mo1	0.02632 (18)	0.0338 (2)	0.02849 (18)	−0.00216 (13)	0.00266 (13)	−0.00110 (13)
Mo2	0.0326 (2)	0.0353 (2)	0.02659 (18)	−0.00396 (14)	0.00280 (13)	0.00113 (14)
O1	0.087 (2)	0.0358 (17)	0.0324 (15)	−0.0170 (16)	0.0071 (14)	0.0005 (13)
O2	0.068 (2)	0.070 (2)	0.056 (2)	−0.0065 (19)	0.0225 (17)	−0.0079 (18)
O3	0.114 (3)	0.069 (3)	0.077 (3)	−0.019 (2)	0.045 (2)	0.011 (2)
O4	0.0321 (16)	0.059 (2)	0.079 (2)	−0.0001 (15)	0.0124 (15)	−0.0006 (18)
O5	0.0436 (18)	0.054 (2)	0.065 (2)	−0.0148 (15)	0.0003 (14)	0.0098 (16)
O6	0.097 (3)	0.0415 (19)	0.060 (2)	0.0189 (19)	0.0004 (18)	0.0078 (17)
O7	0.0340 (18)	0.096 (3)	0.064 (2)	−0.0204 (18)	0.0043 (15)	−0.013 (2)
O8	0.069 (2)	0.068 (2)	0.082 (2)	−0.035 (2)	0.0067 (19)	0.002 (2)
O9	0.065 (2)	0.071 (2)	0.066 (2)	0.0199 (19)	0.0177 (18)	−0.0081 (18)
N1	0.081 (3)	0.041 (2)	0.045 (2)	−0.003 (2)	−0.0002 (19)	−0.0025 (18)
C1	0.103 (4)	0.051 (3)	0.042 (3)	0.002 (3)	−0.006 (3)	−0.002 (2)
C2	0.082 (4)	0.068 (4)	0.036 (2)	0.015 (3)	0.004 (2)	0.010 (2)
C3	0.061 (3)	0.053 (3)	0.043 (2)	0.009 (2)	0.012 (2)	0.013 (2)
C4	0.043 (2)	0.037 (2)	0.052 (3)	0.0070 (19)	0.0159 (19)	0.006 (2)
C5	0.063 (3)	0.037 (3)	0.057 (3)	−0.008 (2)	0.024 (2)	0.004 (2)
C6	0.065 (3)	0.046 (3)	0.055 (3)	−0.022 (2)	0.021 (2)	−0.009 (2)
C7	0.058 (3)	0.040 (2)	0.039 (2)	−0.006 (2)	0.0091 (19)	0.0008 (19)
C8	0.044 (2)	0.036 (2)	0.045 (2)	0.0004 (19)	0.0085 (18)	0.0023 (19)
C9	0.040 (2)	0.043 (2)	0.039 (2)	0.0036 (19)	0.0080 (17)	0.0019 (19)
C10	0.058 (3)	0.039 (2)	0.034 (2)	−0.009 (2)	0.0075 (19)	−0.0002 (18)
C11	0.048 (2)	0.031 (2)	0.0258 (18)	−0.0006 (17)	0.0082 (16)	−0.0001 (16)
C12	0.035 (2)	0.034 (2)	0.0272 (18)	0.0073 (17)	0.0079 (15)	0.0027 (16)
C13	0.039 (2)	0.045 (2)	0.0285 (19)	0.0023 (18)	0.0006 (16)	−0.0038 (17)
C14	0.051 (3)	0.053 (3)	0.027 (2)	−0.012 (2)	0.0098 (18)	−0.0007 (19)
C15	0.035 (2)	0.061 (3)	0.036 (2)	−0.001 (2)	0.0116 (17)	−0.007 (2)
C16	0.041 (2)	0.039 (2)	0.036 (2)	0.0017 (18)	0.0019 (17)	−0.0083 (18)
C17	0.030 (2)	0.040 (2)	0.0289 (19)	−0.0018 (17)	−0.0011 (15)	−0.0052 (16)
C18	0.038 (2)	0.044 (3)	0.035 (2)	−0.009 (2)	0.0008 (17)	−0.0040 (18)
C19	0.058 (3)	0.078 (4)	0.077 (4)	−0.029 (3)	0.004 (3)	0.022 (3)
C20	0.055 (3)	0.054 (3)	0.032 (2)	0.004 (2)	−0.0038 (19)	0.003 (2)
C21	0.041 (2)	0.073 (3)	0.037 (2)	0.006 (2)	−0.0046 (19)	0.001 (2)
C22	0.041 (2)	0.072 (3)	0.040 (2)	−0.015 (2)	−0.0030 (18)	−0.006 (2)
C23	0.051 (3)	0.050 (3)	0.034 (2)	−0.008 (2)	−0.0023 (18)	−0.006 (2)
C24	0.053 (3)	0.052 (3)	0.026 (2)	−0.004 (2)	−0.0028 (18)	0.0025 (18)
C25	0.064 (3)	0.061 (3)	0.030 (2)	−0.013 (3)	0.008 (2)	0.003 (2)
C26	0.076 (4)	0.101 (5)	0.075 (4)	−0.003 (4)	0.027 (3)	−0.004 (4)
C27	0.045 (2)	0.043 (3)	0.039 (2)	−0.007 (2)	0.0052 (18)	0.0046 (19)
C28	0.046 (3)	0.045 (3)	0.034 (2)	−0.004 (2)	0.0077 (18)	0.0006 (18)
C29	0.045 (2)	0.046 (3)	0.037 (2)	0.003 (2)	0.0044 (18)	−0.003 (2)
C30	0.035 (2)	0.055 (3)	0.037 (2)	−0.001 (2)	0.0045 (18)	−0.003 (2)

### Geometric parameters (Å, °)

**Table:**

Mo1—C29	1.949 (5)	C4—C5	1.402 (6)
Mo1—C30	1.967 (4)	C4—C9	1.407 (6)
Mo1—C12	2.108 (4)	C5—C6	1.358 (6)
Mo1—C11	2.198 (4)	C5—H5	0.9300
Mo1—C15	2.334 (4)	C6—C7	1.388 (6)
Mo1—C14	2.341 (4)	C6—H6	0.9300
Mo1—C13	2.349 (4)	C7—C8	1.360 (6)
Mo1—C17	2.355 (4)	C7—H7	0.9300
Mo1—C16	2.360 (4)	C8—C9	1.433 (5)
Mo1—Mo2	2.9778 (5)	C10—C11	1.494 (5)
Mo2—C27	1.998 (4)	C10—H10A	0.9700
Mo2—C28	1.999 (5)	C10—H10B	0.9700
Mo2—C11	2.152 (4)	C11—C12	1.351 (5)
Mo2—C12	2.181 (3)	C12—H12	0.9800
Mo2—C24	2.267 (4)	C13—C14	1.403 (6)
Mo2—C20	2.300 (4)	C13—C17	1.416 (6)
Mo2—C23	2.309 (4)	C13—H13	0.9800
Mo2—C21	2.376 (4)	C14—C15	1.420 (6)
Mo2—C22	2.395 (4)	C14—H14	0.9801
Mo2—C29	2.925 (4)	C15—C16	1.408 (6)
O1—C8	1.343 (5)	C15—H15	0.9800
O1—C10	1.443 (5)	C16—C17	1.421 (5)
O2—C25	1.332 (6)	C16—H16	0.9800
O2—C26	1.444 (6)	C17—C18	1.467 (5)
O3—C25	1.184 (6)	C19—H19A	0.9600
O4—C18	1.200 (5)	C19—H19B	0.9600
O5—C18	1.329 (5)	C19—H19C	0.9600
O5—C19	1.443 (5)	C20—C21	1.401 (6)
O6—C29	1.166 (5)	C20—C24	1.424 (6)
O7—C30	1.132 (5)	C20—H20	0.9800
O8—C27	1.141 (5)	C21—C22	1.399 (7)
O9—C28	1.135 (5)	C21—H21	0.9799
N1—C1	1.318 (6)	C22—C23	1.407 (6)
N1—C9	1.364 (5)	C22—H22	0.9800
C1—C2	1.407 (7)	C23—C24	1.429 (6)
C1—H1	0.9300	C23—H23	0.9801
C2—C3	1.337 (7)	C24—C25	1.480 (6)
C2—H2	0.9300	C26—H26A	0.9600
C3—C4	1.416 (6)	C26—H26B	0.9600
C3—H3	0.9300	C26—H26C	0.9600
			
C29—Mo1—C30	89.90 (18)	C6—C5—H5	120.3
C29—Mo1—C12	110.20 (15)	C4—C5—H5	120.3
C30—Mo1—C12	108.77 (16)	C5—C6—C7	121.5 (4)
C29—Mo1—C11	112.88 (15)	C5—C6—H6	119.2
C30—Mo1—C11	72.37 (16)	C7—C6—H6	119.2
C12—Mo1—C11	36.50 (14)	C8—C7—C6	121.0 (4)
C29—Mo1—C15	102.97 (16)	C8—C7—H7	119.5
C30—Mo1—C15	93.99 (15)	C6—C7—H7	119.5
C12—Mo1—C15	139.24 (14)	O1—C8—C7	125.9 (4)
C11—Mo1—C15	141.19 (15)	O1—C8—C9	114.9 (4)
C29—Mo1—C14	138.27 (17)	C7—C8—C9	119.2 (4)
C30—Mo1—C14	94.36 (16)	N1—C9—C4	123.4 (4)
C12—Mo1—C14	107.56 (15)	N1—C9—C8	118.0 (4)
C11—Mo1—C14	107.93 (15)	C4—C9—C8	118.7 (4)
C15—Mo1—C14	35.35 (15)	O1—C10—C11	104.8 (3)
C29—Mo1—C13	138.59 (16)	O1—C10—H10A	110.8
C30—Mo1—C13	125.06 (16)	C11—C10—H10A	110.8
C12—Mo1—C13	81.27 (14)	O1—C10—H10B	110.8
C11—Mo1—C13	99.77 (14)	C11—C10—H10B	110.8
C15—Mo1—C13	58.06 (14)	H10A—C10—H10B	108.9
C14—Mo1—C13	34.82 (14)	C12—C11—C10	133.7 (4)
C29—Mo1—C17	103.70 (16)	C12—C11—Mo2	73.0 (2)
C30—Mo1—C17	150.95 (14)	C10—C11—Mo2	135.7 (3)
C12—Mo1—C17	90.70 (14)	C12—C11—Mo1	68.2 (2)
C11—Mo1—C17	122.87 (14)	C10—C11—Mo1	132.8 (3)
C15—Mo1—C17	58.24 (13)	Mo2—C11—Mo1	86.40 (14)
C14—Mo1—C17	58.47 (14)	C11—C12—Mo1	75.3 (2)
C13—Mo1—C17	35.05 (14)	C11—C12—Mo2	70.7 (2)
C29—Mo1—C16	84.98 (16)	Mo1—C12—Mo2	87.91 (14)
C30—Mo1—C16	124.01 (15)	C11—C12—H12	132.9
C12—Mo1—C16	125.27 (14)	Mo1—C12—H12	132.9
C11—Mo1—C16	157.05 (14)	Mo2—C12—H12	132.9
C15—Mo1—C16	34.90 (14)	C14—C13—C17	108.9 (4)
C14—Mo1—C16	58.56 (15)	C14—C13—Mo1	72.3 (2)
C13—Mo1—C16	58.18 (14)	C17—C13—Mo1	72.7 (2)
C17—Mo1—C16	35.09 (13)	C14—C13—H13	125.4
C29—Mo1—Mo2	69.26 (12)	C17—C13—H13	125.4
C30—Mo1—Mo2	86.48 (12)	Mo1—C13—H13	125.4
C12—Mo1—Mo2	47.06 (9)	C13—C14—C15	107.2 (4)
C11—Mo1—Mo2	46.17 (9)	C13—C14—Mo1	72.9 (2)
C15—Mo1—Mo2	172.23 (11)	C15—C14—Mo1	72.0 (2)
C14—Mo1—Mo2	152.38 (11)	C13—C14—H14	126.2
C13—Mo1—Mo2	127.49 (10)	C15—C14—H14	126.2
C17—Mo1—Mo2	122.23 (9)	Mo1—C14—H14	126.2
C16—Mo1—Mo2	140.55 (10)	C16—C15—C14	108.8 (4)
C27—Mo2—C28	83.48 (18)	C16—C15—Mo1	73.5 (2)
C27—Mo2—C11	116.18 (15)	C14—C15—Mo1	72.6 (2)
C28—Mo2—C11	87.96 (15)	C16—C15—H15	125.4
C27—Mo2—C12	80.38 (15)	C14—C15—H15	125.4
C28—Mo2—C12	77.29 (14)	Mo1—C15—H15	125.4
C11—Mo2—C12	36.32 (14)	C15—C16—C17	107.5 (4)
C27—Mo2—C24	97.27 (16)	C15—C16—Mo1	71.6 (2)
C28—Mo2—C24	88.75 (16)	C17—C16—Mo1	72.3 (2)
C11—Mo2—C24	145.74 (16)	C15—C16—H16	126.1
C12—Mo2—C24	166.01 (15)	C17—C16—H16	126.1
C27—Mo2—C20	91.10 (17)	Mo1—C16—H16	126.1
C28—Mo2—C20	123.69 (15)	C13—C17—C16	107.5 (3)
C11—Mo2—C20	141.41 (16)	C13—C17—C18	124.5 (4)
C12—Mo2—C20	156.56 (15)	C16—C17—C18	128.0 (4)
C24—Mo2—C20	36.31 (15)	C13—C17—Mo1	72.2 (2)
C27—Mo2—C23	131.81 (15)	C16—C17—Mo1	72.6 (2)
C28—Mo2—C23	83.32 (16)	C18—C17—Mo1	120.9 (2)
C11—Mo2—C23	109.40 (15)	O4—C18—O5	124.1 (4)
C12—Mo2—C23	140.08 (16)	O4—C18—C17	123.9 (4)
C24—Mo2—C23	36.38 (15)	O5—C18—C17	112.0 (4)
C20—Mo2—C23	59.77 (16)	O5—C19—H19A	109.5
C27—Mo2—C21	118.77 (17)	O5—C19—H19B	109.5
C28—Mo2—C21	140.90 (16)	H19A—C19—H19B	109.5
C11—Mo2—C21	106.79 (16)	O5—C19—H19C	109.5
C12—Mo2—C21	134.52 (14)	H19A—C19—H19C	109.5
C24—Mo2—C21	58.59 (16)	H19B—C19—H19C	109.5
C20—Mo2—C21	34.81 (15)	C21—C20—C24	107.3 (4)
C23—Mo2—C21	57.72 (16)	C21—C20—Mo2	75.6 (3)
C27—Mo2—C22	149.46 (17)	C24—C20—Mo2	70.6 (2)
C28—Mo2—C22	112.41 (17)	C21—C20—H20	126.1
C11—Mo2—C22	91.16 (16)	C24—C20—H20	126.1
C12—Mo2—C22	127.40 (16)	Mo2—C20—H20	126.1
C24—Mo2—C22	58.97 (16)	C22—C21—C20	109.8 (4)
C20—Mo2—C22	58.36 (17)	C22—C21—Mo2	73.7 (3)
C23—Mo2—C22	34.74 (15)	C20—C21—Mo2	69.6 (2)
C21—Mo2—C22	34.09 (17)	C22—C21—H21	125.1
C27—Mo2—C29	74.82 (15)	C20—C21—H21	125.1
C28—Mo2—C29	150.76 (14)	Mo2—C21—H21	125.1
C11—Mo2—C29	84.39 (13)	C21—C22—C23	107.5 (4)
C12—Mo2—C29	79.92 (12)	C21—C22—Mo2	72.2 (2)
C24—Mo2—C29	112.95 (14)	C23—C22—Mo2	69.3 (2)
C20—Mo2—C29	76.77 (14)	C21—C22—H22	126.2
C23—Mo2—C29	125.81 (14)	C23—C22—H22	126.2
C21—Mo2—C29	68.09 (14)	Mo2—C22—H22	126.2
C22—Mo2—C29	95.96 (14)	C22—C23—C24	108.2 (4)
C27—Mo2—Mo1	86.85 (11)	C22—C23—Mo2	76.0 (2)
C28—Mo2—Mo1	122.32 (11)	C24—C23—Mo2	70.2 (2)
C11—Mo2—Mo1	47.43 (10)	C22—C23—H23	125.7
C12—Mo2—Mo1	45.03 (9)	C24—C23—H23	125.7
C24—Mo2—Mo1	148.92 (12)	Mo2—C23—H23	125.7
C20—Mo2—Mo1	113.18 (11)	C20—C24—C23	107.2 (4)
C23—Mo2—Mo1	138.11 (11)	C20—C24—C25	125.3 (4)
C21—Mo2—Mo1	92.41 (11)	C23—C24—C25	127.5 (4)
C22—Mo2—Mo1	104.12 (11)	C20—C24—Mo2	73.1 (2)
C29—Mo2—Mo1	38.54 (8)	C23—C24—Mo2	73.4 (2)
C8—O1—C10	118.2 (3)	C25—C24—Mo2	118.4 (3)
C25—O2—C26	115.9 (4)	O3—C25—O2	124.7 (5)
C18—O5—C19	115.3 (4)	O3—C25—C24	125.0 (5)
C1—N1—C9	116.8 (4)	O2—C25—C24	110.2 (4)
N1—C1—C2	123.9 (5)	O2—C26—H26A	109.5
N1—C1—H1	118.1	O2—C26—H26B	109.5
C2—C1—H1	118.1	H26A—C26—H26B	109.5
C3—C2—C1	119.4 (4)	O2—C26—H26C	109.5
C3—C2—H2	120.3	H26A—C26—H26C	109.5
C1—C2—H2	120.3	H26B—C26—H26C	109.5
C2—C3—C4	119.9 (4)	O8—C27—Mo2	177.6 (4)
C2—C3—H3	120.1	O9—C28—Mo2	176.3 (4)
C4—C3—H3	120.1	O6—C29—Mo1	169.9 (4)
C5—C4—C9	120.1 (4)	O6—C29—Mo2	117.8 (3)
C5—C4—C3	123.2 (4)	Mo1—C29—Mo2	72.20 (13)
C9—C4—C3	116.7 (4)	O7—C30—Mo1	177.2 (4)
C6—C5—C4	119.5 (4)		
			
C29—Mo1—Mo2—C27	69.40 (18)	C11—Mo1—C15—C16	141.8 (3)
C30—Mo1—Mo2—C27	160.62 (18)	C14—Mo1—C15—C16	116.4 (4)
C12—Mo1—Mo2—C27	−79.54 (19)	C13—Mo1—C15—C16	78.9 (3)
C11—Mo1—Mo2—C27	−130.58 (19)	C17—Mo1—C15—C16	37.4 (2)
C14—Mo1—Mo2—C27	−106.7 (3)	C29—Mo1—C15—C14	−177.3 (3)
C13—Mo1—Mo2—C27	−66.68 (18)	C30—Mo1—C15—C14	91.9 (3)
C17—Mo1—Mo2—C27	−24.08 (17)	C12—Mo1—C15—C14	−33.2 (4)
C16—Mo1—Mo2—C27	16.80 (19)	C11—Mo1—C15—C14	25.4 (4)
C29—Mo1—Mo2—C28	149.6 (2)	C13—Mo1—C15—C14	−37.5 (2)
C30—Mo1—Mo2—C28	−119.13 (19)	C17—Mo1—C15—C14	−79.0 (3)
C12—Mo1—Mo2—C28	0.7 (2)	C16—Mo1—C15—C14	−116.4 (4)
C11—Mo1—Mo2—C28	−50.3 (2)	C14—C15—C16—C17	0.7 (4)
C14—Mo1—Mo2—C28	−26.4 (3)	Mo1—C15—C16—C17	−63.8 (3)
C13—Mo1—Mo2—C28	13.57 (19)	C14—C15—C16—Mo1	64.5 (3)
C17—Mo1—Mo2—C28	56.17 (18)	C29—Mo1—C16—C15	121.3 (3)
C16—Mo1—Mo2—C28	97.1 (2)	C30—Mo1—C16—C15	34.9 (3)
C29—Mo1—Mo2—C11	−160.0 (2)	C12—Mo1—C16—C15	−127.4 (2)
C30—Mo1—Mo2—C11	−68.80 (19)	C11—Mo1—C16—C15	−96.2 (4)
C12—Mo1—Mo2—C11	51.0 (2)	C14—Mo1—C16—C15	−37.4 (2)
C14—Mo1—Mo2—C11	23.9 (3)	C13—Mo1—C16—C15	−78.5 (3)
C13—Mo1—Mo2—C11	63.91 (19)	C17—Mo1—C16—C15	−116.1 (3)
C17—Mo1—Mo2—C11	106.51 (18)	Mo2—Mo1—C16—C15	169.55 (19)
C16—Mo1—Mo2—C11	147.4 (2)	C29—Mo1—C16—C17	−122.6 (3)
C29—Mo1—Mo2—C12	148.9 (2)	C30—Mo1—C16—C17	150.9 (2)
C30—Mo1—Mo2—C12	−119.84 (19)	C12—Mo1—C16—C17	−11.3 (3)
C11—Mo1—Mo2—C12	−51.0 (2)	C11—Mo1—C16—C17	19.9 (5)
C14—Mo1—Mo2—C12	−27.2 (3)	C15—Mo1—C16—C17	116.1 (3)
C13—Mo1—Mo2—C12	12.86 (19)	C14—Mo1—C16—C17	78.7 (3)
C17—Mo1—Mo2—C12	55.47 (19)	C13—Mo1—C16—C17	37.6 (2)
C16—Mo1—Mo2—C12	96.3 (2)	Mo2—Mo1—C16—C17	−74.4 (3)
C29—Mo1—Mo2—C24	−29.5 (3)	C14—C13—C17—C16	0.8 (4)
C30—Mo1—Mo2—C24	61.7 (3)	Mo1—C13—C17—C16	64.5 (2)
C12—Mo1—Mo2—C24	−178.4 (3)	C14—C13—C17—C18	−179.5 (3)
C11—Mo1—Mo2—C24	130.5 (3)	Mo1—C13—C17—C18	−115.7 (3)
C14—Mo1—Mo2—C24	154.4 (3)	C14—C13—C17—Mo1	−63.7 (3)
C13—Mo1—Mo2—C24	−165.6 (3)	C15—C16—C17—C13	−0.9 (4)
C17—Mo1—Mo2—C24	−123.0 (2)	Mo1—C16—C17—C13	−64.3 (2)
C16—Mo1—Mo2—C24	−82.1 (3)	C15—C16—C17—C18	179.3 (3)
C29—Mo1—Mo2—C20	−20.45 (19)	Mo1—C16—C17—C18	116.0 (4)
C30—Mo1—Mo2—C20	70.77 (18)	C15—C16—C17—Mo1	63.3 (3)
C12—Mo1—Mo2—C20	−169.39 (19)	C29—Mo1—C17—C13	175.4 (2)
C11—Mo1—Mo2—C20	139.57 (19)	C30—Mo1—C17—C13	59.6 (4)
C14—Mo1—Mo2—C20	163.5 (3)	C12—Mo1—C17—C13	−73.6 (2)
C13—Mo1—Mo2—C20	−156.53 (18)	C11—Mo1—C17—C13	−55.3 (3)
C17—Mo1—Mo2—C20	−113.93 (17)	C15—Mo1—C17—C13	78.4 (3)
C16—Mo1—Mo2—C20	−73.0 (2)	C14—Mo1—C17—C13	36.6 (2)
C29—Mo1—Mo2—C23	−90.4 (2)	C16—Mo1—C17—C13	115.6 (3)
C30—Mo1—Mo2—C23	0.8 (2)	Mo2—Mo1—C17—C13	−110.7 (2)
C12—Mo1—Mo2—C23	120.7 (2)	C29—Mo1—C17—C16	59.8 (3)
C11—Mo1—Mo2—C23	69.6 (2)	C30—Mo1—C17—C16	−56.0 (4)
C14—Mo1—Mo2—C23	93.5 (3)	C12—Mo1—C17—C16	170.8 (2)
C13—Mo1—Mo2—C23	133.5 (2)	C11—Mo1—C17—C16	−170.9 (2)
C17—Mo1—Mo2—C23	176.1 (2)	C15—Mo1—C17—C16	−37.2 (2)
C16—Mo1—Mo2—C23	−143.0 (2)	C14—Mo1—C17—C16	−79.0 (3)
C29—Mo1—Mo2—C21	−49.30 (18)	C13—Mo1—C17—C16	−115.6 (3)
C30—Mo1—Mo2—C21	41.93 (18)	Mo2—Mo1—C17—C16	133.7 (2)
C12—Mo1—Mo2—C21	161.76 (19)	C29—Mo1—C17—C18	−64.6 (3)
C11—Mo1—Mo2—C21	110.72 (19)	C30—Mo1—C17—C18	179.7 (3)
C14—Mo1—Mo2—C21	134.6 (3)	C12—Mo1—C17—C18	46.4 (3)
C13—Mo1—Mo2—C21	174.63 (18)	C11—Mo1—C17—C18	64.8 (4)
C17—Mo1—Mo2—C21	−142.77 (17)	C15—Mo1—C17—C18	−161.5 (4)
C16—Mo1—Mo2—C21	−101.9 (2)	C14—Mo1—C17—C18	156.7 (4)
C29—Mo1—Mo2—C22	−81.72 (19)	C13—Mo1—C17—C18	120.1 (4)
C30—Mo1—Mo2—C22	9.50 (18)	C16—Mo1—C17—C18	−124.3 (4)
C12—Mo1—Mo2—C22	129.3 (2)	Mo2—Mo1—C17—C18	9.4 (4)
C11—Mo1—Mo2—C22	78.3 (2)	C19—O5—C18—O4	1.2 (6)
C14—Mo1—Mo2—C22	102.2 (3)	C19—O5—C18—C17	179.6 (3)
C13—Mo1—Mo2—C22	142.21 (18)	C13—C17—C18—O4	−5.7 (6)
C17—Mo1—Mo2—C22	−175.19 (18)	C16—C17—C18—O4	174.0 (4)
C16—Mo1—Mo2—C22	−134.3 (2)	Mo1—C17—C18—O4	−94.5 (4)
C30—Mo1—Mo2—C29	91.22 (18)	C13—C17—C18—O5	175.9 (3)
C12—Mo1—Mo2—C29	−148.9 (2)	C16—C17—C18—O5	−4.4 (5)
C11—Mo1—Mo2—C29	160.0 (2)	Mo1—C17—C18—O5	87.0 (4)
C14—Mo1—Mo2—C29	−176.1 (3)	C27—Mo2—C20—C21	−144.7 (3)
C13—Mo1—Mo2—C29	−136.08 (19)	C28—Mo2—C20—C21	132.5 (3)
C17—Mo1—Mo2—C29	−93.48 (18)	C11—Mo2—C20—C21	−7.6 (4)
C16—Mo1—Mo2—C29	−52.6 (2)	C12—Mo2—C20—C21	−76.7 (5)
C9—N1—C1—C2	0.4 (8)	C24—Mo2—C20—C21	114.5 (4)
N1—C1—C2—C3	−0.4 (9)	C23—Mo2—C20—C21	75.8 (3)
C1—C2—C3—C4	0.5 (8)	C22—Mo2—C20—C21	35.1 (3)
C2—C3—C4—C5	179.3 (5)	C29—Mo2—C20—C21	−70.5 (3)
C2—C3—C4—C9	−0.6 (7)	Mo1—Mo2—C20—C21	−57.6 (3)
C9—C4—C5—C6	−2.8 (7)	C27—Mo2—C20—C24	100.8 (3)
C3—C4—C5—C6	177.2 (4)	C28—Mo2—C20—C24	17.9 (3)
C4—C5—C6—C7	2.2 (7)	C11—Mo2—C20—C24	−122.2 (3)
C5—C6—C7—C8	0.0 (7)	C12—Mo2—C20—C24	168.7 (3)
C10—O1—C8—C7	−0.1 (6)	C23—Mo2—C20—C24	−38.7 (3)
C10—O1—C8—C9	178.8 (4)	C21—Mo2—C20—C24	−114.5 (4)
C6—C7—C8—O1	177.3 (4)	C22—Mo2—C20—C24	−79.4 (3)
C6—C7—C8—C9	−1.5 (7)	C29—Mo2—C20—C24	174.9 (3)
C1—N1—C9—C4	−0.5 (7)	Mo1—Mo2—C20—C24	−172.1 (2)
C1—N1—C9—C8	178.8 (4)	C24—C20—C21—C22	0.9 (5)
C5—C4—C9—N1	−179.3 (4)	Mo2—C20—C21—C22	−63.1 (3)
C3—C4—C9—N1	0.6 (6)	C24—C20—C21—Mo2	64.0 (3)
C5—C4—C9—C8	1.3 (6)	C27—Mo2—C21—C22	160.3 (2)
C3—C4—C9—C8	−178.7 (4)	C28—Mo2—C21—C22	42.3 (4)
O1—C8—C9—N1	2.5 (6)	C11—Mo2—C21—C22	−65.9 (3)
C7—C8—C9—N1	−178.6 (4)	C12—Mo2—C21—C22	−93.8 (3)
O1—C8—C9—C4	−178.1 (4)	C24—Mo2—C21—C22	79.9 (3)
C7—C8—C9—C4	0.8 (6)	C20—Mo2—C21—C22	119.0 (4)
C8—O1—C10—C11	−179.1 (3)	C23—Mo2—C21—C22	36.8 (2)
O1—C10—C11—C12	59.7 (6)	C29—Mo2—C21—C22	−142.5 (3)
O1—C10—C11—Mo2	173.4 (3)	Mo1—Mo2—C21—C22	−111.9 (2)
O1—C10—C11—Mo1	−40.9 (5)	C27—Mo2—C21—C20	41.3 (3)
C27—Mo2—C11—C12	−10.6 (3)	C28—Mo2—C21—C20	−76.8 (4)
C28—Mo2—C11—C12	71.1 (2)	C11—Mo2—C21—C20	175.0 (3)
C24—Mo2—C11—C12	155.9 (3)	C12—Mo2—C21—C20	147.1 (3)
C20—Mo2—C11—C12	−141.1 (3)	C24—Mo2—C21—C20	−39.1 (3)
C23—Mo2—C11—C12	153.3 (2)	C23—Mo2—C21—C20	−82.3 (3)
C21—Mo2—C11—C12	−145.7 (2)	C22—Mo2—C21—C20	−119.0 (4)
C22—Mo2—C11—C12	−176.5 (2)	C29—Mo2—C21—C20	98.4 (3)
C29—Mo2—C11—C12	−80.6 (2)	Mo1—Mo2—C21—C20	129.0 (3)
Mo1—Mo2—C11—C12	−68.2 (2)	C20—C21—C22—C23	−0.1 (5)
C27—Mo2—C11—C10	−146.8 (4)	Mo2—C21—C22—C23	−60.6 (3)
C28—Mo2—C11—C10	−65.1 (4)	C20—C21—C22—Mo2	60.6 (3)
C12—Mo2—C11—C10	−136.2 (5)	C27—Mo2—C22—C21	−35.5 (4)
C24—Mo2—C11—C10	19.7 (6)	C28—Mo2—C22—C21	−152.7 (3)
C20—Mo2—C11—C10	82.6 (5)	C11—Mo2—C22—C21	119.0 (3)
C23—Mo2—C11—C10	17.1 (5)	C12—Mo2—C22—C21	116.4 (3)
C21—Mo2—C11—C10	78.1 (4)	C24—Mo2—C22—C21	−78.7 (3)
C22—Mo2—C11—C10	47.3 (4)	C20—Mo2—C22—C21	−35.9 (3)
C29—Mo2—C11—C10	143.2 (4)	C23—Mo2—C22—C21	−117.3 (4)
Mo1—Mo2—C11—C10	155.5 (5)	C29—Mo2—C22—C21	34.6 (3)
C27—Mo2—C11—Mo1	57.68 (19)	Mo1—Mo2—C22—C21	72.9 (3)
C28—Mo2—C11—Mo1	139.39 (15)	C27—Mo2—C22—C23	81.8 (4)
C12—Mo2—C11—Mo1	68.2 (2)	C28—Mo2—C22—C23	−35.4 (3)
C24—Mo2—C11—Mo1	−135.8 (2)	C11—Mo2—C22—C23	−123.6 (3)
C20—Mo2—C11—Mo1	−72.9 (3)	C12—Mo2—C22—C23	−126.3 (3)
C23—Mo2—C11—Mo1	−138.42 (14)	C24—Mo2—C22—C23	38.6 (3)
C21—Mo2—C11—Mo1	−77.44 (16)	C20—Mo2—C22—C23	81.4 (3)
C22—Mo2—C11—Mo1	−108.23 (16)	C21—Mo2—C22—C23	117.3 (4)
C29—Mo2—C11—Mo1	−12.35 (12)	C29—Mo2—C22—C23	151.9 (3)
C29—Mo1—C11—C12	93.4 (2)	Mo1—Mo2—C22—C23	−169.8 (3)
C30—Mo1—C11—C12	175.6 (3)	C21—C22—C23—C24	−0.8 (5)
C15—Mo1—C11—C12	−110.6 (3)	Mo2—C22—C23—C24	−63.3 (3)
C14—Mo1—C11—C12	−95.5 (2)	C21—C22—C23—Mo2	62.5 (3)
C13—Mo1—C11—C12	−60.6 (2)	C27—Mo2—C23—C22	−137.6 (3)
C17—Mo1—C11—C12	−31.9 (3)	C28—Mo2—C23—C22	147.4 (3)
C16—Mo1—C11—C12	−45.4 (4)	C11—Mo2—C23—C22	61.9 (3)
Mo2—Mo1—C11—C12	73.1 (2)	C12—Mo2—C23—C22	86.4 (4)
C29—Mo1—C11—C10	−136.5 (4)	C24—Mo2—C23—C22	−115.6 (4)
C30—Mo1—C11—C10	−54.3 (4)	C20—Mo2—C23—C22	−77.0 (3)
C12—Mo1—C11—C10	130.1 (5)	C21—Mo2—C23—C22	−36.1 (3)
C15—Mo1—C11—C10	19.5 (5)	C29—Mo2—C23—C22	−35.3 (3)
C14—Mo1—C11—C10	34.6 (4)	Mo1—Mo2—C23—C22	14.9 (4)
C13—Mo1—C11—C10	69.5 (4)	C27—Mo2—C23—C24	−22.0 (4)
C17—Mo1—C11—C10	98.2 (4)	C28—Mo2—C23—C24	−97.0 (3)
C16—Mo1—C11—C10	84.7 (5)	C11—Mo2—C23—C24	177.5 (3)
Mo2—Mo1—C11—C10	−156.8 (5)	C12—Mo2—C23—C24	−158.0 (3)
C29—Mo1—C11—Mo2	20.30 (19)	C20—Mo2—C23—C24	38.6 (3)
C30—Mo1—C11—Mo2	102.47 (17)	C21—Mo2—C23—C24	79.5 (3)
C12—Mo1—C11—Mo2	−73.1 (2)	C22—Mo2—C23—C24	115.6 (4)
C15—Mo1—C11—Mo2	176.28 (16)	C29—Mo2—C23—C24	80.3 (3)
C14—Mo1—C11—Mo2	−168.62 (13)	Mo1—Mo2—C23—C24	130.5 (2)
C13—Mo1—C11—Mo2	−133.69 (13)	C21—C20—C24—C23	−1.3 (5)
C17—Mo1—C11—Mo2	−105.06 (15)	Mo2—C20—C24—C23	66.0 (3)
C16—Mo1—C11—Mo2	−118.5 (3)	C21—C20—C24—C25	179.5 (4)
C10—C11—C12—Mo1	−129.0 (4)	Mo2—C20—C24—C25	−113.1 (4)
Mo2—C11—C12—Mo1	92.97 (11)	C21—C20—C24—Mo2	−67.3 (3)
C10—C11—C12—Mo2	138.0 (4)	C22—C23—C24—C20	1.3 (5)
Mo1—C11—C12—Mo2	−92.97 (11)	Mo2—C23—C24—C20	−65.8 (3)
C29—Mo1—C12—C11	−101.5 (2)	C22—C23—C24—C25	−179.6 (4)
C30—Mo1—C12—C11	−4.4 (3)	Mo2—C23—C24—C25	113.3 (4)
C15—Mo1—C12—C11	116.0 (3)	C22—C23—C24—Mo2	67.1 (3)
C14—Mo1—C12—C11	96.6 (2)	C27—Mo2—C24—C20	−81.9 (3)
C13—Mo1—C12—C11	119.7 (2)	C28—Mo2—C24—C20	−165.2 (3)
C17—Mo1—C12—C11	153.6 (2)	C11—Mo2—C24—C20	110.3 (3)
C16—Mo1—C12—C11	160.1 (2)	C12—Mo2—C24—C20	−161.3 (6)
Mo2—Mo1—C12—C11	−70.6 (2)	C23—Mo2—C24—C20	114.4 (4)
C29—Mo1—C12—Mo2	−30.94 (19)	C21—Mo2—C24—C20	37.5 (3)
C30—Mo1—C12—Mo2	66.12 (17)	C22—Mo2—C24—C20	77.6 (3)
C11—Mo1—C12—Mo2	70.6 (2)	C29—Mo2—C24—C20	−5.4 (3)
C15—Mo1—C12—Mo2	−173.43 (17)	Mo1—Mo2—C24—C20	14.1 (4)
C14—Mo1—C12—Mo2	167.18 (13)	C27—Mo2—C24—C23	163.7 (3)
C13—Mo1—C12—Mo2	−169.70 (15)	C28—Mo2—C24—C23	80.4 (3)
C17—Mo1—C12—Mo2	−135.82 (14)	C11—Mo2—C24—C23	−4.1 (4)
C16—Mo1—C12—Mo2	−129.33 (14)	C12—Mo2—C24—C23	84.3 (7)
C27—Mo2—C12—C11	170.4 (3)	C20—Mo2—C24—C23	−114.4 (4)
C28—Mo2—C12—C11	−104.2 (2)	C21—Mo2—C24—C23	−76.9 (3)
C24—Mo2—C12—C11	−108.2 (6)	C22—Mo2—C24—C23	−36.8 (3)
C20—Mo2—C12—C11	100.4 (4)	C29—Mo2—C24—C23	−119.8 (3)
C23—Mo2—C12—C11	−41.3 (3)	Mo1—Mo2—C24—C23	−100.3 (3)
C21—Mo2—C12—C11	49.2 (3)	C27—Mo2—C24—C25	39.6 (4)
C22—Mo2—C12—C11	4.4 (3)	C28—Mo2—C24—C25	−43.7 (4)
C29—Mo2—C12—C11	94.3 (2)	C11—Mo2—C24—C25	−128.2 (4)
Mo1—Mo2—C12—C11	75.2 (2)	C12—Mo2—C24—C25	−39.8 (8)
C27—Mo2—C12—Mo1	95.19 (16)	C20—Mo2—C24—C25	121.5 (5)
C28—Mo2—C12—Mo1	−179.39 (17)	C23—Mo2—C24—C25	−124.1 (5)
C11—Mo2—C12—Mo1	−75.2 (2)	C21—Mo2—C24—C25	159.0 (4)
C24—Mo2—C12—Mo1	176.6 (6)	C22—Mo2—C24—C25	−161.0 (4)
C20—Mo2—C12—Mo1	25.2 (4)	C29—Mo2—C24—C25	116.1 (4)
C23—Mo2—C12—Mo1	−116.5 (2)	Mo1—Mo2—C24—C25	135.6 (3)
C21—Mo2—C12—Mo1	−26.0 (3)	C26—O2—C25—O3	4.7 (7)
C22—Mo2—C12—Mo1	−70.8 (2)	C26—O2—C25—C24	−174.6 (4)
C29—Mo2—C12—Mo1	19.06 (12)	C20—C24—C25—O3	−1.3 (7)
C29—Mo1—C13—C14	110.2 (3)	C23—C24—C25—O3	179.8 (5)
C30—Mo1—C13—C14	−32.2 (3)	Mo2—C24—C25—O3	−90.0 (6)
C12—Mo1—C13—C14	−139.0 (3)	C20—C24—C25—O2	178.1 (4)
C11—Mo1—C13—C14	−107.4 (3)	C23—C24—C25—O2	−0.9 (6)
C15—Mo1—C13—C14	38.1 (2)	Mo2—C24—C25—O2	89.4 (4)
C17—Mo1—C13—C14	117.1 (4)	C30—Mo1—C29—O6	101 (2)
C16—Mo1—C13—C14	79.4 (3)	C12—Mo1—C29—O6	−149 (2)
Mo2—Mo1—C13—C14	−148.5 (2)	C11—Mo1—C29—O6	171 (2)
C29—Mo1—C13—C17	−6.8 (4)	C15—Mo1—C29—O6	7(2)
C30—Mo1—C13—C17	−149.2 (2)	C14—Mo1—C29—O6	4(2)
C12—Mo1—C13—C17	103.9 (2)	C13—Mo1—C29—O6	−49 (2)
C11—Mo1—C13—C17	135.5 (2)	C17—Mo1—C29—O6	−53 (2)
C15—Mo1—C13—C17	−79.0 (3)	C16—Mo1—C29—O6	−24 (2)
C14—Mo1—C13—C17	−117.1 (4)	Mo2—Mo1—C29—O6	−173 (2)
C16—Mo1—C13—C17	−37.6 (2)	C30—Mo1—C29—Mo2	−86.28 (13)
Mo2—Mo1—C13—C17	94.4 (2)	C12—Mo1—C29—Mo2	23.73 (14)
C17—C13—C14—C15	−0.3 (4)	C11—Mo1—C29—Mo2	−15.52 (15)
Mo1—C13—C14—C15	−64.3 (3)	C15—Mo1—C29—Mo2	179.65 (10)
C17—C13—C14—Mo1	64.0 (3)	C14—Mo1—C29—Mo2	177.28 (18)
C29—Mo1—C14—C13	−111.2 (3)	C13—Mo1—C29—Mo2	123.67 (19)
C30—Mo1—C14—C13	154.1 (3)	C17—Mo1—C29—Mo2	119.64 (10)
C12—Mo1—C14—C13	42.8 (3)	C16—Mo1—C29—Mo2	149.55 (11)
C11—Mo1—C14—C13	81.2 (3)	C27—Mo2—C29—O6	74.2 (4)
C15—Mo1—C14—C13	−115.2 (4)	C28—Mo2—C29—O6	117.7 (4)
C17—Mo1—C14—C13	−36.9 (2)	C11—Mo2—C29—O6	−166.7 (4)
C16—Mo1—C14—C13	−78.3 (3)	C12—Mo2—C29—O6	156.9 (4)
Mo2—Mo1—C14—C13	63.3 (4)	C24—Mo2—C29—O6	−17.4 (4)
C29—Mo1—C14—C15	4.0 (4)	C20—Mo2—C29—O6	−20.6 (4)
C30—Mo1—C14—C15	−90.7 (3)	C23—Mo2—C29—O6	−56.8 (4)
C12—Mo1—C14—C15	158.0 (2)	C21—Mo2—C29—O6	−56.1 (4)
C11—Mo1—C14—C15	−163.6 (2)	C22—Mo2—C29—O6	−76.1 (4)
C13—Mo1—C14—C15	115.2 (4)	Mo1—Mo2—C29—O6	178.6 (4)
C17—Mo1—C14—C15	78.3 (3)	C27—Mo2—C29—Mo1	−104.43 (16)
C16—Mo1—C14—C15	36.9 (2)	C28—Mo2—C29—Mo1	−60.9 (3)
Mo2—Mo1—C14—C15	178.51 (18)	C11—Mo2—C29—Mo1	14.65 (14)
C13—C14—C15—C16	−0.3 (4)	C12—Mo2—C29—Mo1	−21.76 (13)
Mo1—C14—C15—C16	−65.1 (3)	C24—Mo2—C29—Mo1	163.99 (14)
C13—C14—C15—Mo1	64.9 (3)	C20—Mo2—C29—Mo1	160.74 (17)
C29—Mo1—C15—C16	−60.8 (3)	C23—Mo2—C29—Mo1	124.57 (16)
C30—Mo1—C15—C16	−151.6 (3)	C21—Mo2—C29—Mo1	125.28 (18)
C12—Mo1—C15—C16	83.3 (3)	C22—Mo2—C29—Mo1	105.23 (15)

### Hydrogen-bond geometry (Å, °)

**Table:**

*D*—H···*A*	*D*—H	H···*A*	*D*···*A*	*D*—H···*A*
C14—H14···N1	0.98	2.61	3.344 (6)	132
C15—H15···O4^i^	0.98	2.36	3.191 (5)	143

Symmetry codes: (i) *x*−1, *y*, *z*.
